# The Antiglaucoma Agent and EP2 Receptor Agonist Omidenepag Does Not Affect Eyelash Growth in Mice

**DOI:** 10.1089/jop.2020.0003

**Published:** 2020-09-07

**Authors:** Yoshihiko Esaki, Osamu Katsuta, Hitomi Kamio, Takahisa Noto, Hidetoshi Mano, Ryo Iwamura, Kenji Yoneda, Noriko Odani-Kawabata, Kenji Morishima, Naveed K. Shams

**Affiliations:** ^1^ Santen Pharmaceutical Co., Ltd., Research and Development Division, Nara, Japan.; ^2^ Ube Industries, Ltd., Pharmaceuticals Research Laboratory, Pharmaceutical Division, Yamaguchi, Japan.; ^3^ Santen Pharmaceutical Co., Ltd., Osaka, Japan.; ^4^ Santen, Inc., Research and Development Division, Emeryville, California, USA.

**Keywords:** EP2 agonist, omidenepag, FP agonist, eyelash growth

## Abstract

***Purpose:*** The present study investigated the effects of the antiglaucoma agent and selective E2 receptor agonist omidenepag isopropyl (OMDI) on eyelash growth in comparison with a prostaglandin analog (prostamide receptor agonist) in mice.

***Methods:*** Four-week-old female mice (C57BL/6J) were divided into 3 groups of *n* = 10 each. The groups were administered 3 μL of 0.003% OMDI solution, the vehicle (negative control), or a 0.03% bimatoprost solution (positive control) on the upper eyelids of the right eyes once daily for 14 days. On the 15th day, all animals were euthanized, and the upper eyelids with eyelashes were fixed with 10% neutral formalin. Eyelashes were evaluated for number, length, and thickness using a stereomicroscope. Specimens were then paraffin-embedded and stained with hematoxylin and eosin, followed by microscopic examination to assess eyelash morphology and growth cycle.

***Results:*** Eyelash number (143.5 ± 6.7/eyelid), thickness, and percentage of dermal papilla in the anagen phase in the OMDI group were similar to those observed in the vehicle group (eyelash number, 144.2 ± 5.7/eyelid). In contrast, eyelash number (166.7 ± 7.0/eyelid), thickness, and the percentage of dermal papilla in the anagen phase were significantly greater in the bimatoprost group compared with those of the vehicle group.

***Conclusions:*** Unlike existing prostaglandin analogs, our findings indicate that OMDI has no effect on eyelash growth in mice, suggesting that it may be a promising antiglaucoma agent with a reduced number of adverse effects.

## Introduction

Worldwide, glaucoma is a major ocular disorder characterized by progressive optic neuropathy and the loss of retinal ganglion cells and their axons, resulting in visual field defects.^1–3^ Elevated intraocular pressure (IOP) is an important risk factor for glaucoma and, to date, lowering IOP is the only treatment strategy that has been shown to slow or stop the structural and functional progression of glaucoma.^4^ As the current standard of care, prostaglandin F_2α_ receptor agonists (FP agonists), such as latanoprost, tafluprost, and travoprost, and prostamide receptor agonists, such as bimatoprost, are used to lower IOP in patients with glaucoma.^5,6^ However, in these patients, the occurrence of prostaglandin-associated periorbitopathy, including abnormal eyelash growth, has been reported.^7,8^ This eyelash growth can cause cosmetic events in these patients.^9–11^ For example, case reports of abnormal eyelash growth caused by long-term use of bimatoprost in patients with glaucoma have been reported.^9,12^ Bimatoprost has also been reported to promote eyelash growth by increasing the growing phase (i.e., the anagen phase) of the follicles in mice.^13^ Although the detailed molecular mechanisms regulating the rate of eyelash growth remain unclear, these previous studies have suggested that abnormal eyelash growth caused by prostamide or FP agonists may be due to the dysregulation of the eyelash growth rate and hair growth cycle.

Prostaglandin E_2_ (PGE_2_) lowers IOP by acting on a group of G protein-coupled receptors.^14^ Thus, PGE_2_ receptor agonists as ocular hypotensive agents are being actively investigated.^15^

Omidenepag isopropyl (OMDI) is the prodrug of a selective, nonprostaglandin EP2 receptor agonist and is being developed as a new IOP-lowering topical agent worldwide.^16–18^ In 2018, OMDI was approved and launched for glaucoma and ocular hypertension in Japan. During corneal penetration, OMDI is hydrolyzed to omidenepag (OMD) by esterases, which results in its IOP-lowering effects by increasing outflow facility and uveoscleral outflow.^19^ In the associated clinical study, OMDI demonstrated stable IOP-lowering effects and was well tolerated for 3 months.^18^ The topical application of 0.002% OMDI resulted in clinically significant IOP reduction in patients with glaucoma for 12 months (Aihara, et al.)^18^. However, its adverse reactions in humans are now being investigated further through postmarketing surveillance.

To date, the effects of the EP2 agonist OMDI on eyelash growth has not been determined. Therefore, the present study was conducted to investigate the effects of OMDI on eyelash growth in comparison with a prostaglandin analog (prostamide receptor agonist) in mice.

## Methods

This research followed the Association for Research in Vision and Ophthalmology for the Use of Animals in Ophthalmic and Vision Research, and the research was approved by the Animal Experiment Committee of Santen Pharmaceutical Co., Ltd. (Approval No. DR-2016-0214).

## Materials

OMDI was provided by Ube Industries Ltd. (Yamaguchi, Japan). The structure of OMDI has been described previously.^17^ A dosing solution of 0.003% OMDI was formulated by Santen Pharmaceutical Co., Ltd. (Osaka, Japan).

### Animals

Four-week-old female mice (C57BL/6J, 13.1–15.9 g before dosing start) were purchased (Japan SLC, Inc., Japan) and divided into 3 groups of *n* = 10 each for the administration of 0.003% OMDI, saline (Otsuka Normal Saline; Otsuka Pharmaceutical Factory, Inc., Tokushima, Japan), or 0.03% bimatoprost (BIM) ophthalmic solution (Lumigan^®^ 0.03%; Senju Pharmaceutical Co., Ltd., Osaka, Japan). The mice had access to food and water *ad libitum* and were housed for 27 days, including a quarantine and acclimation period for 13 days and a dosing period for 14 days, in animal cages with a 12 h light/dark cycle under controlled conditions of temperature (20°C–26°C) and humidity (30%–70%). This study was reviewed by the Animal Care and Use Committee and approved by the director of the associated institution (the animal experiment committee of Santen Pharmaceutical Co., Ltd.; Approval No. DR-2016-0214). All protocols were conducted in accordance with the Association for Research in Vision and Ophthalmology statement for the use of animals in ophthalmic and vision research and animal welfare bylaws of Santen Pharmaceutical Co., Ltd., Nara Research and Development Center, Japan.

This mouse model is considered reproducible, quantifiable, and pragmatic for studies on eyelash growth.^13^

### Administration and tissue sampling

Animals were administered 3 μL of 0.003% OMDI solution, saline (Otsuka Normal Saline; Otsuka Pharmaceutical Factory, Inc.) as a negative control, or a 0.03% BIM ophthalmic solution (Lumigan 0.03%; Senju Pharmaceutical Co., Ltd.) as a positive control on the right upper eyelids once daily for 14 days. The left upper eyelids remained untreated.

On the day following the end of dosing, all animals were euthanized by inhalation anesthesia of carbon dioxide, after which the right upper eyelid was extracted and fixed with 10% formalin neutral buffer solution.

### Determination of eyelash number and classification

From the temporal corner to the nasal corner of the eyelid, the number of eyelashes was counted in each animal using a stereoscopic microscope. The eyelash length was classified into 3 categories as follows^13^:

- Short: ≤250 μm
- Middle: 250–450 μm
- Long: 450–2,500 μm

### Measuring the eyelash thickness

After classifying the length, the thickness of each eyelash was measured using a scale capable of 0.1 μm accuracy.

### Histopathological observation

The eyelids were then embedded in paraffin, and 3 μm thick serial cross sections (*n* = 50) stained with hematoxylin and eosin were prepared according to the Standard Operating Procedures of the test facility (Santen Pharmaceutical Co., Ltd., Nara Research and Development Center). The growth cycle of the eyelash bulb papillae was classified for each treatment group by microscopic evaluation according to the characterization of the eyelash growth phase.^13^

### Statistical analysis

Eyelash number and thickness are expressed as the mean ± standard deviation for each group. Statistical analyses were conducted using EXSUS (CAC Croit Corporation, Japan). Student's *t*-test was performed to test the significance of differences between the drug treatment and control groups. The hair growth cycle was expressed as the percentage of each hair growth cycle. The *z*-test was performed to test the significance of the differences between the drug treatment and control groups. *P* < 0.05 was considered statistically significant.

## Results

### Determination of eyelash number and classification

**FIG. 1.**
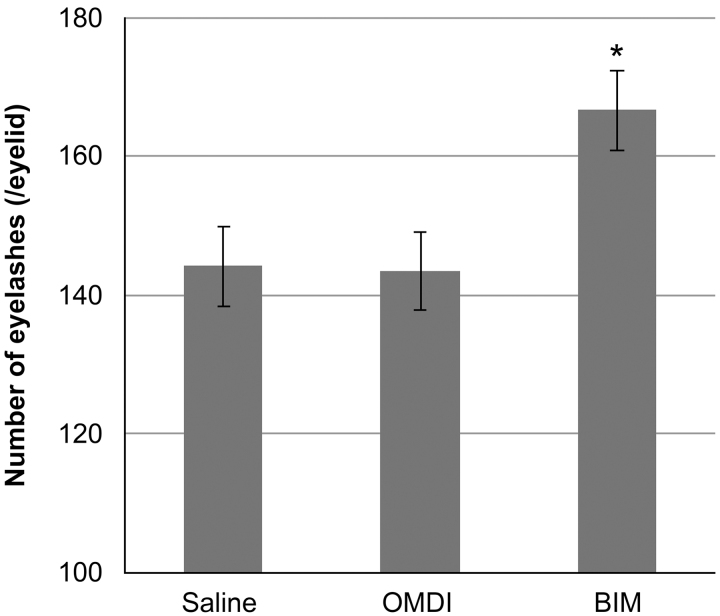
Number of eyelashes after the 14-day dosing period in each group. From the inner corner to the outer corner of the eye, the number of eyelashes was counted in each animal using a stereoscopic microscope. Comparison of eyelash number between control (saline) and OMDI and BIM groups was performed by Student's *t*-test. *P < 0.05, compared with control. OMDI, omidenepag isopropyl.

The mean numbers of eyelashes/eyelid were 144 ± 6, 144 ± 7, and 167 ± 7 for the saline, OMDI, and BIM groups, respectively. OMDI did not change the number of eyelashes (*P* = 0.795 vs. saline). In contrast, a significant increase in eyelash number is observed in the BIM group (*P* < 0.05 vs. saline) (Fig. 1).

### Measuring eyelash thickness

**FIG. 2.**
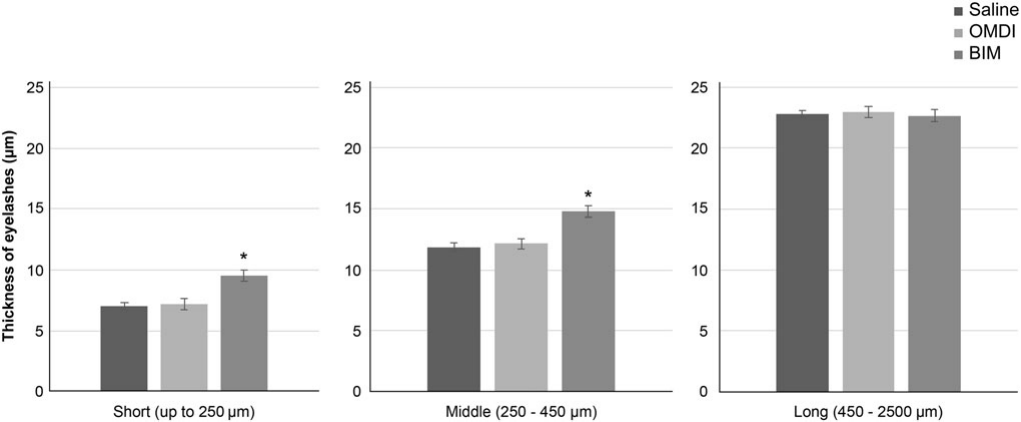
Eyelash thickness after the 14-day dosing period in each group. The eyelash length was classified into 3 categories as follows: Short (up to 250 μm), middle (250–450 μm), and long (450–2,500 μm); after classifying, the thickness of each was measured by using a scale capable of 0.1 μm accuracy. Comparison of eyelash thickness between control (saline) and OMDI and BIM was performed by Student's *t*-test. *P < 0.05, compared with control.

The mean eyelash thickness according to length in the OMDI group was not significantly different from that of the saline group. For the BIM group, the thicknesses of eyelashes of short and middle length were significantly increased compared with the saline group (Fig. 2). The mean eyelash thicknesses of short-length eyelashes (up to 250 μm) were 7.0 ± 0.3, 7.2 ± 0.4, and 9.5 ± 0.5 μm for the saline, OMDI, and BIM groups, respectively. The mean eyelash thicknesses of middle-length eyelashes (250–450 μm) were 11.9 ± 0.6, 12.2 ± 0.8, and 14.8 ± 0.6 μm for the saline, OMDI, and BIM groups, respectively. There were no differences in the mean eyelash thicknesses of long-length eyelashes (450–2,500 μm) among the groups. For long-length eyelashes, the mean eyelash thicknesses were 22.8 ± 0.9, 23.0 ± 0.5, and 22.7 ± 0.9 μm for the saline, OMDI, and BIM groups, respectively.

### Histopathological observation

**FIG. 3.**
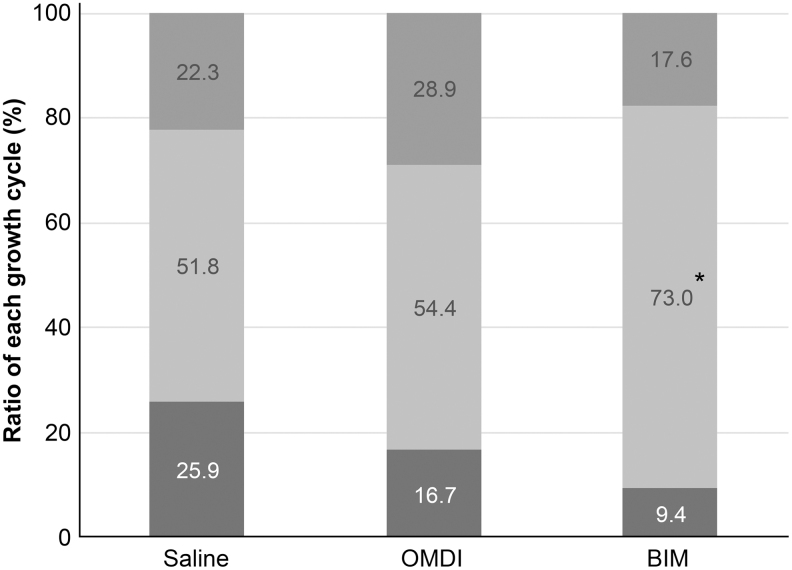
Classification of the growth cycle in each group. The growth cycle of eyelash bulb papillae was classified in each treatment group by microscopic evaluation. The graph shows the growth cycle (starting from *top*): catagen phase, anagen phase, and telogen phase. Comparison between growth cycle of control (saline) and OMDI and BIM was performed by *z*-test. *P < 0.05, compared with control.

**FIG. 4.**
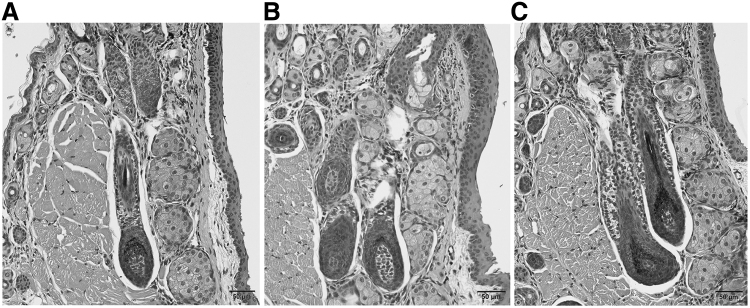
Histopathological observation of bulb papillae in each group. Typical bulb papillae in anagen phase **(A)** in saline control group, **(B)** in OMDI group, **(C)** in BIM group. Eyelash bulb papillae in the anagen phase in the BIM group were thicker than those observed for either the saline or OMDI group.

For the OMDI group, the percentage of eyelash papillae in the anagen phase and the growing phase of the hair cycle were not significantly different from the saline group (OMDI: 54.4%; saline: 51.8%). For the BIM group (73%), the percentage of eyelash papillae in the anagen phase was significantly increased compared with that in the saline group (51.8%; *P* < 0.05 vs. saline) (Fig. 3). From the histopathological examination, eyelash bulb papillae in the anagen phase in the BIM group were thicker than those observed for either the saline or OMDI group (Fig. 4).

## Discussion

In the present study, we investigated the effects of a novel selective EP2 agonist, OMDI, on eyelash growth in mice. The C57BL/6J mouse model used in this study has been studied in detail regarding the eyelash hair cycle.^13^ Our results clarified that OMDI, with its active metabolite OMD, is a selective, nonprostaglandin prostanoid EP2 receptor (EP2) agonist and has no effect on eyelash number (Fig. 1), thickness (Fig. 2), or the percentage of eyelash papillae in the anagen phase (Fig. 3). In contrast, the BIM-positive control affected all of these parameters. From histopathological analysis, the number of eyelash bulb papillae in the anagen phase in the OMDI group was similar to that for the saline group. In contrast, the eyelash bulb papillae in the BIM group were thicker compared with the saline group (Fig. 4).

In patients with glaucoma and ocular hypertension treated with the prostaglandin analogs, including bimatoprost, the occurrence of abnormal eyelash growth on the eyelid has been reported.^7,20,21^ The findings of this study also confirmed that bimatoprost promoted eyelash growth by increasing the number of growing (anagen phase) follicles in mice, as previously reported by Tauchi et al.^13^

Abnormal eyelash growth by FP agonists may be caused by a combination of several factors. In human hair follicles, PGF_2α_ receptors are located predominantly in the inner root sheath of the bulb and stem of eyelashes and expressed only in eyelashes in the anagen phase.^22^ The prolongation of the anagen phase through actions targeting the hair papilla cells in the early anagen phase may be directly caused by FP receptor agonists. Secondary effects of FP receptor agonists, such as vasodilation, cell growth promotion, stimulation of cell adhesion molecule expression, extracellular matrix remodeling, and increased nutrient metabolism activity via increased intracellular calcium ion concentrations, may also contribute to the growth of the hair follicles.^23,24^ While this study does not show the relationships between these mechanisms and EP2 receptor agonists, OMDI is a nonprostaglandin structure compound with a high affinity for the EP2 receptor and almost no affinity for the FP receptor.^17^ Our results indicate that OMDI does not affect the eyelash growth cycle and does not promote eyelash growth, which is in contrast to FP receptor agonists in mice in the growth cycle tested in these experiments.

Our current results are consistent with those observed in humans administered a 52-week treatment regimen of 0.002% OMDI eye drops, which had no effect on eyelash growth (Aihara, et al.).^18^ In 2018, OMDI was approved and launched for glaucoma and ocular hypertension in Japan. This property of OMDI, which does not promote eyelash growth in mice, may provide an option of treatment for glaucoma and ocular hypertension without eyelash growth.

In conclusion, our data indicated that OMDI did not promote eyelash growth in mice, while bimatoprost did. Collectively, these findings suggest that OMDI may be a promising antiglaucoma agent with a reduced number of adverse effects. The results of a postmarketing survey for patients treated with OMDI for a longer duration are expected in the near future may clarify whether OMDI promotes eyelash growth in human after a long treatment duration.
